# Overweight with HBV infection limited the efficacy of TACE in hepatocellular carcinoma by inhibiting the upregulated HMGB1

**DOI:** 10.1186/s12885-021-08783-8

**Published:** 2021-09-28

**Authors:** Yuan-dong Sun, Hao Zhang, Ye-qiang Chen, Chun-xue Wu, Miao-ling Chen, Hui-rong Xu, Shuo Wang, Jing-zhou Liu, Jian-jun Han

**Affiliations:** 1 Interventional Radiology Department, Shandong Cancer Hospital and Institute Affiliated Shandong First Medical University and Shandong Academy of Medical Sciences, No. 440, Jiyan Road, Jinan, 250117 Shandong Province China; 2 Maternal and Child Health Care Hospital of Shandong Province, No 238, Jingshidong Raod, Jinan, 250014 Shandong Province China; 3 Shandong First Medical University, No. 6699, Qingdao Road, Jinan, 250062 Shandong Province China

**Keywords:** Hepatocellular carcinomas, Body mass index, Hepatitis B virus, Transarterial chemoembolization, HMGB1

## Abstract

**Background:**

Transarterial chemoembolization (TACE) is an effective treatment for patients with hepatocellular carcinoma (HCC). However, the impact of hepatitis B viral (HBV) infection and body mass index (BMI) on TACE is controversial. The present study aimed to compare the influence of HBV and high BMI on TACE outcomes in advanced HCC.

**Methods:**

Based on HBV infection history and BMI, patients were assigned to different subgroups. Blood samples were collected and analyzed by an enzyme-linked immunosorbent assay (ELISA) kit. The primary endpoint was progression-free survival (PFS) and the overall survival (OS) in the population.

**Results:**

Compared to overweight combined HBV patients who received TACE, people with normal weight or no viral infection had significantly better OS and PFS. Sex, age, portal vein tumor thrombus, BCLC, ECOG, and tumor diameter are the main risk factors affecting PFS and OS. Except for the postoperative fever, no significant difference was detected in adverse reactions. Irrespective of TACE, the average expression of HMGB1 in hepatitis or obesity patients was higher than that in normal individuals and did not show upregulation after TACE. Patients without overweight or HBV infection had a low expression of serum HMGB1 that was substantially upregulated after TACE.

**Conclusions:**

In this study, overweight combined HBV infection patients had shorter PFS and OS than other HCC patients. Thus, HBV and BMI maybe two factors affecting the efficacy of TACE via upregulated HMGB1.

## Highlight

1. High BMI combined with HBV infection has a negative impact on the PFS and OS of HCC patients treated with TACE.

2. Irrespective of TACE, the average HMGB1 in hepatitis and obesity patients was higher than that in normal individuals, which was not upregulated after TACE.

3. Patients without overweight and HBV infection had a low level of serum concentration of HMGB1 before TACE that was upregulated after TACE. Compared to others, these patients have severe changes in HMGB1 in the peripheral blood after treatment, indicating a higher risk of acute liver injury.

4. Upregulated HMGB1 elevated the prognosis after TACE, but obesity and HBV infection resulted in the negative effect by inhibiting HMGB1 expression.

5. For advanced HCC, overweight combined HBV infection, PVTT, multiple lesions, and larger tumor diameter are negative factors for TACE.

## Background

Primary liver cancer is one of the highest mortality cancers of the digestive system in Asia. It is also the third most common cause of cancer-related deaths worldwide [1]. Although significant differences were noted in etiology, the incidence of hepatocellular carcinoma (HCC) in both developed and developing countries has increased during the past three decades [2, 3]. Based on the results of current etiological studies, scientists found that viral hepatitis, alcohol, obesity, smoking, and aflatoxin are the main risk factors for HCC [4–7]. Although overweight is associated with liver disease and has long been considered a major cause of HCC, our understanding of the role of body mass index (BMI) and hepatitis B virus (HBV) infection in cancer treatment is limited [8–10]. Recent studies have reported that obesity associated with viral infection increases both the risk and the rate of HCC progression. The resulting health issues gained increasing attention [11, 12]. Reportedly, in 2014, more than 1.9 billion adults worldwide were overweight or obese and associated with various liver diseases, from hepatic steatosis, nonalcoholic fatty liver disease (NAFLD) to severe nonalcoholic steatohepatitis (NASH) [13]. With the increase in obesity and the incidence of these diseases, obese patients with HCC have become common.

However, few studies have focused on the impact of high-risk factors on the treatment of advanced HCC. Presently, no study has compared the effects of BMI combined with HBV infection on patients with advanced HCC. Are there any differences in the results of HBV-infected HCC patients with different BMI after the same treatment? Will BMI complicated with HBV infection increase the incidence of adverse reactions? It is necessary to explore the influence of high BMI and HBV infection and possible risk factors on the therapeutic effect of patients.

The basic principle of transarterial chemoembolization (TACE) is the combination of hepatic arterial chemoembolization and arterial embolization. This is one of the standard effective treatments for patients with early or advanced HCC [14]. In this retrospective study, we analyzed the safety and outcomes of 9687 Chinese advanced HCC patients treated with TACE. Subsequently, patients were divided into different groups based on BMI and HBV infection history. All the participants in this study were recruited from our center (Shandong Cancer Hospital). The survival data of this study were collected through the medical record management system and follow-up. The progression-free survival (PFS), overall survival (OS), and adverse reactions of patients in different groups were counted and compared. Peripheral venous blood was withdrawn from patients, and an enzyme-linked immunosorbent assay (ELISA) kit was used to analyze the preliminary changes in human high mobility group 1 (HMGB1)-related pathways and altered expression. We also aimed to evaluate the factors that predict the safety and efficacy of TACE in patients with HBV infection and different weights, including PFS, OS, and adverse reactions, and to establish a basis to construct animal models and pathway studies.

## Methods

### Patients

The Institutional Ethics Committee of Shandong Cancer Hospital and Institute approved retrospective analysis of patients with HCC admitted to the center from January 2012 to December 2017. Only patients who received TACE (chemoembolization with CalliSpheres® microspheres and conventional chemoembolization) were included in the data analysis. Although doctors provided various treatment options, these patients chose TACE as their single anticancer treatment. None of the patients in this study received surgery/immunotherapy during the follow-up period. The Ethical Committee of Shandong Cancer Hospital checked our investigation. This is a anonymous retrospective study, ethical approval was not required.

Pre-TACE examination includes HBV serological examination and liver function test in all patients. Computed tomography (CT) and/or magnetic resonance imaging (MRI) were performed to evaluate the degree of disease and the location and characteristics of the tumor.

Based on the patient’s medical records, we selected HCC patients caused by HBV cirrhosis and defined them as HBV patients.

Overweight was defined as BMI > 23 kg/m^2^ according to the Asian and Chinese criteria.

**Fig. 1:**
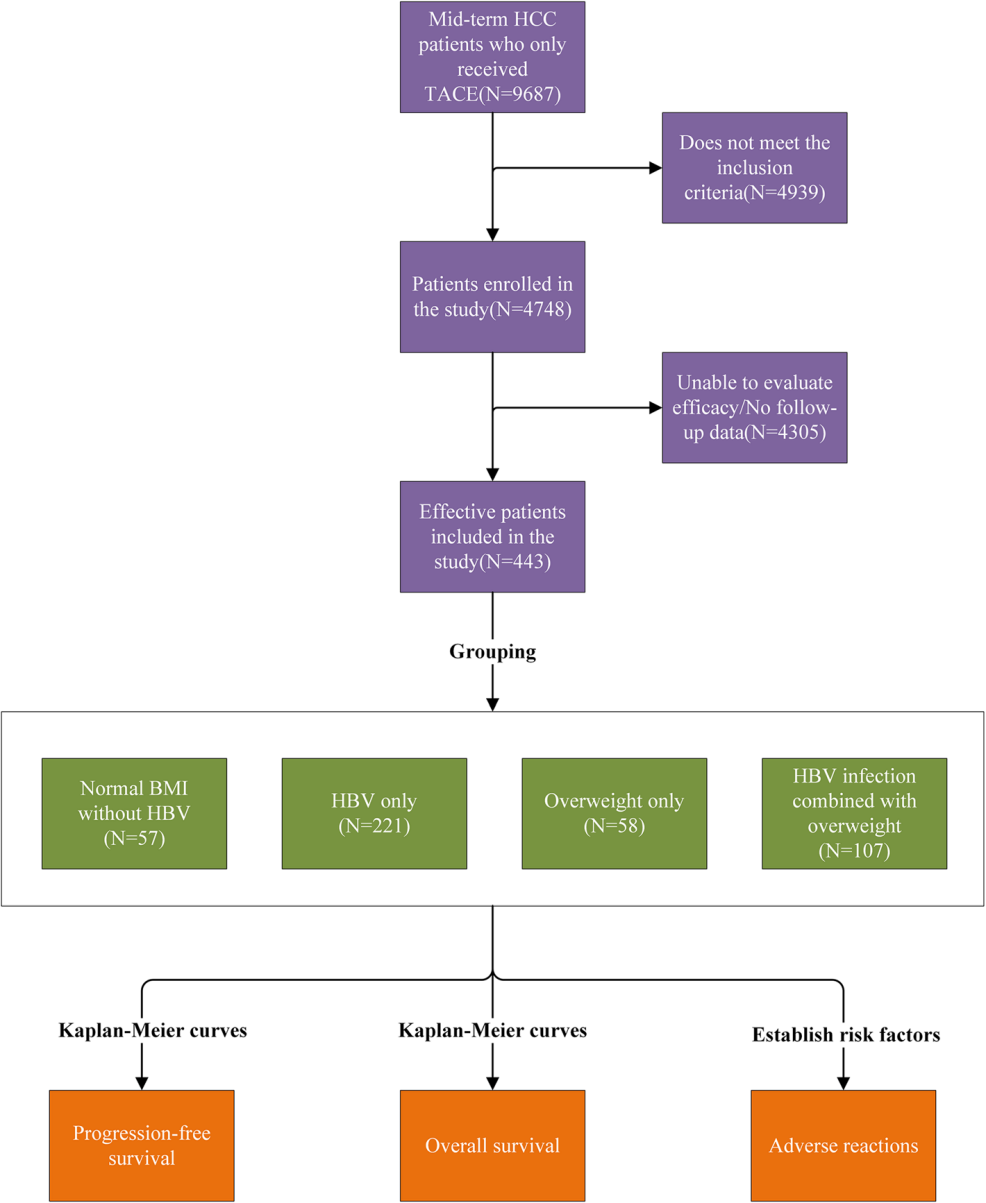
The flowchart of this study. Cohort identification and inclusion/exclusion flow chart in this study. HCC = hepatocellular carcinoma; TACE = transarterial chemoembolization; HBV = hepatitis B virus; BMI = body mass index

The patients in this study were divided into four subgroups (Table 1). Primary analysis was stratified by age, sex, Child–Pugh score, BCLC staging system, Milan criteria, radiology data (number and size of tumors), the efficacy of treatment (PFS, OS, and tumor response), and common adverse reactions after TACE. Patients in both groups received super-selective TACE. No artificial control was observed between chemotherapeutic drugs and fixed materials. The follow-up program included regular follow-up, with contrast-enhanced abdominal computed tomography (CT)/magnetic resonance imaging (MRI) every 2 months for the first year and every 2–3 months subsequently. The endpoints of follow-up were death and progression of the disease. The end of the follow-up was in June 2020. The whole process of this study is illustrated in Fig. 1.

**Table 1:** Definition of patients in each subgroup

Subgroup	Patient characteristics
Group 1	Normal weight patients without HBV infection
Group 2	Normal weight patients combined HBV infection
Group 3	Overweight patients without HBV infection
Group 4	Overweight patients combined HBV infection

### Serum markers measurement

After fasting for at least 8 h, blood samples were collected from the anterior elbow vein by centrifugation at 1000×*g* for 10 min and stored at − 80 °C until further analysis. According to the manufacturer’s instructions, the serum concentrations of HMGB1, receptor of advanced glycation end products (RAGE), tumor necrosis factor-alpha (TNF-α), and vascular endothelial growth factor (VEGF) were quantified using an ELISA kit (NOVUS Biologicals, Littleton, CO,USA).

### Statistical analysis

All statistical analyses were carried out using SPSS version 24.0 software (SPSS Inc., Chicago, IL, USA). The data are reported as numbers (percentage) and median (range), except for age, expressed as mean ± standard deviation (SD). Tumor response results were evaluated according to the Modified Response Evaluation Criteria in Solid Tumors (mRECIST), and the patient’s baseline characteristics were assessed based on the qualitative variables by χ^2^ and Fisher’s text. PFS and OS are estimated by Kaplan–Meier method. A logarithmic rank test was used for comparison (for univariate analysis in each group). Multivariate analysis used an input Cox proportional hazard model and included variables with *P*-values < 0.10 from univariate analysis. *P* < 0.05 indicated statistical significance.

## Results

### Patient data analysis

A total of 443 patients with HCC were included in this study, of which 328 (74.04%) had HBV infection, and 165 (37.25%) were obese. The patients with HBV (56.91 ± 9.04 years) were younger than the overweight individuals (59.27 ± 12.03 years). During the study period, 443 patients underwent 979 TACE processes. The clinical features of the patients are described in Table 2.

**Table 2:** Clinical characteristics of patients

Parameters	Group1^a^ (*n* = 57)	Group2^b^ (*n* = 221)	Group3^c^ (*n* = 58)	Group4^d^ (*n* = 107)	F	***P***
**Age** (mean ± SD)	60.67 ± 12.01	57.12 ± 9.31	57.07 ± 11.12	55.48 ± 9.62	3.342	0.019
**Sex**					2.315	0.075
Male	52	194	44	91		
Female	5	27	14	16		
**PVTT**^e^					0.585	0.625
Yes	11	44	16	23		
No	46	177	42	84		
**BCLC**^f^					0.027	0.994
A	10	27	8	15		
B	32	147	34	68		
C	15	47	16	24		
**Child–Pugh**					3.150	0.025
A	22	41	20	33		
B	35	179	38	74		
C	0	1	0	0		
**Milan criteria**					1.101	0.348
Yes	14	79	20	41		
No	43	142	38	66		
**Total tumor diameter (mm)**					2.740	0.043
< 30	5	43	8	19		
30–50	10	57	14	32		
> 50	42	121	36	56		
**Number of lesions**					3.230	0.22
1	50	161	50	87		
2	1	12	2	6		
≥ 3	6	48	6	14		

^a^Group 1: normal-weight patients without HBV

^b^Group 2: normal-weight patients combined HBV

^c^Group 3: overweight patients without HBV

^d^Group 4: overweight patients combined HBV

^e^*PVTT* Portal vein tumor thrombus

^f^*BCLC* Barcelona Clinic Liver Cancer

A total of 132 blood samples were withdrawn from 33 patients before and after TACE and assessed with ELISA kit. The baseline characteristics are listed in Table 3.

**Table 3:** Clinical characteristics of patients in blood analysis

Parameters	Group 1^a^ (*n* = 4)	Group 2^b^ (*n* = 18)	Group 3^c^ (*n* = 6)	Group 4^d^ (*n* = 5)
Age (mean ± SD)	58.75 ± 12.12	61.05 ± 6.33	55.5 ± 6.16	60 ± 6.36
**Sex**				
Male	3	14	4	3
Female	1	4	2	2
**PVTT**^e^				
Yes	0	6	2	1
No	4	12	4	4
**BCLC**^f^				
A	2	5	2	1
B	2	11	3	3
C	0	2	1	1
**Child–Pugh**				
A	1	4	1	2
B	3	13	5	3
C	0	1	0	0
**Milan criteria**				
**Yes**	1	2	1	1
**No**	3	16	5	4
**Total tumor diameter (mm)**				
**< 30**	1	3	1	0
**30–50**	2	2	3	4
**> 50**	1	13	2	1
**Number of lesions**				
**1**	4	17	5	4
**2**	0	1	1	1
**≥ 3**	0	0	0	0

^a^Group 1: normal-weight patients without HBV

^b^Group 2: normal-weight patients combined HBV

^c^Group 3: overweight patients without HBV

^d^Group 4: overweight patients combined HBV

^e^*PVTT* Portal vein tumor thrombus

^f^*BCLC* Barcelona Clinic Liver Cancer

### Subgroup analysis of PFS and OS

**Fig. 2:**
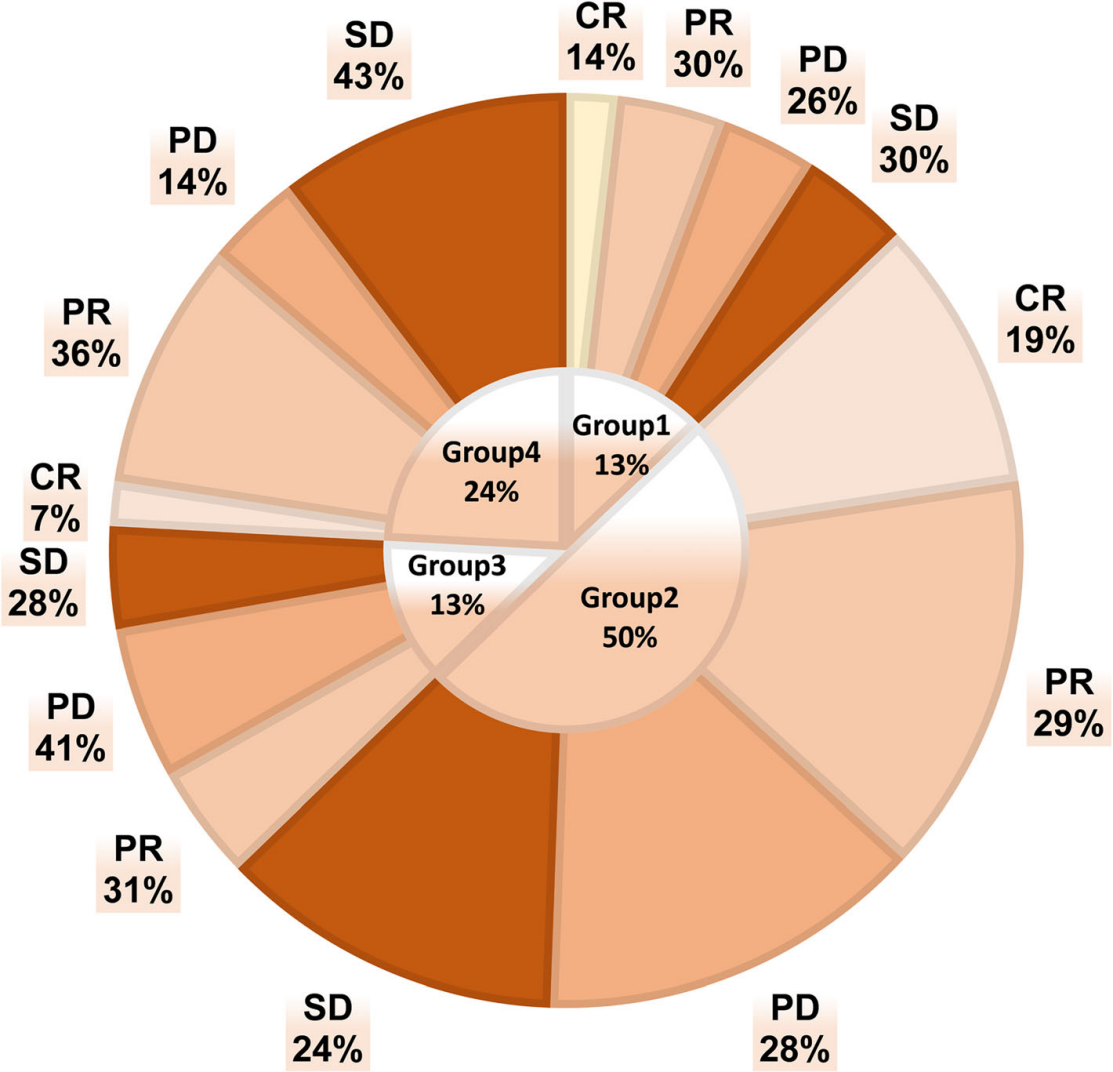
Tumor response after TACE in the four groups of HCC patients. Group1 = Normal weight HCC patients without HBV infection; Group2 = Normal weight HCC patients combined HBV infection; Group3 = Overweight HCC patients without HBV infection; Group4 = Overweight HCC patients combined HBV infection; CR = Complete response; PR = Partial response; SD=Stable disease; PD=Progressive disease

**Fig. 3:**
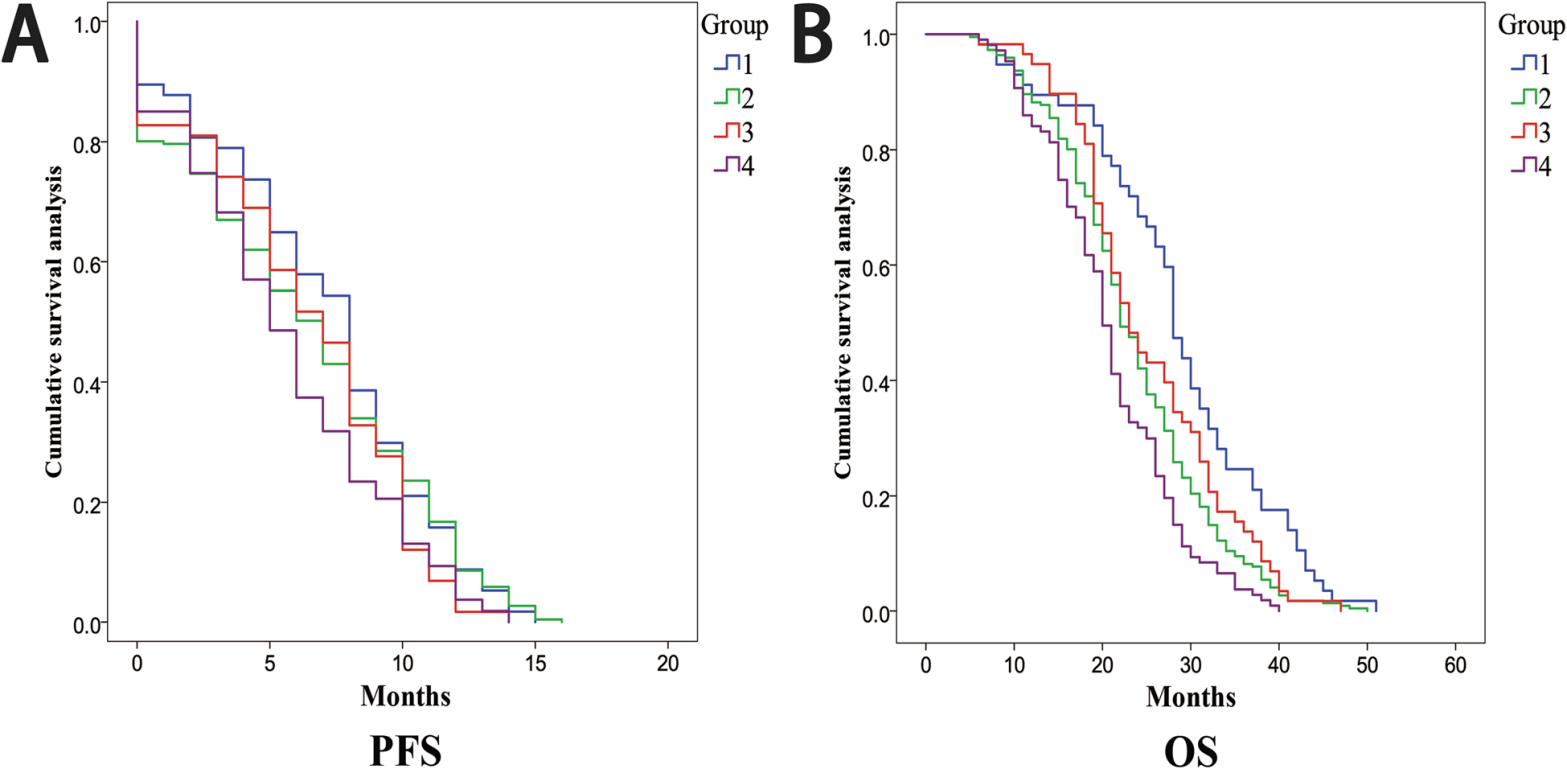
The overall PFS and OS curves show the PFS and OS of all patients reviewed. Group1 = Normal weight HCC patients without HBV infection; Group2 = Normal weight HCC patients combined HBV infection; Group3 = Overweight HCC patients without HBV infection; Group4 = Overweight HCC patients combined HBV infection; PFS = progression-free survival; OS = overall survival

According to the clinical staging analysis, no difference was detected in the Child–Pugh or BCLC staging system among the subgroups (Table 2). A total of 94 (21.22%) patients reported portal vein tumor thrombus (PVTT). The patients included in this study had advanced liver cancer, and the purpose of the treatment was to prolong the survival time. The tumor response of each group is shown in Fig. 2. The analysis of the Kaplan–Meier curve (Fig. 3) revealed that under similar conditions, the PFS and OS of the normal weight patients (Group 1 and Group 2) seem to be better than those of the overweight people in the study (Group 3 and Group 4).

The characteristics of the tumor are listed in Table 2. The tumor diameter and number of lesions were also varied: about 57.56% of the patients had a tumor size of > 50 mm. Three-quarters of patients had a single lesion, while 16.7% had more than two lesions.

### Multiple risk factors of PFS

**Fig. 4:**
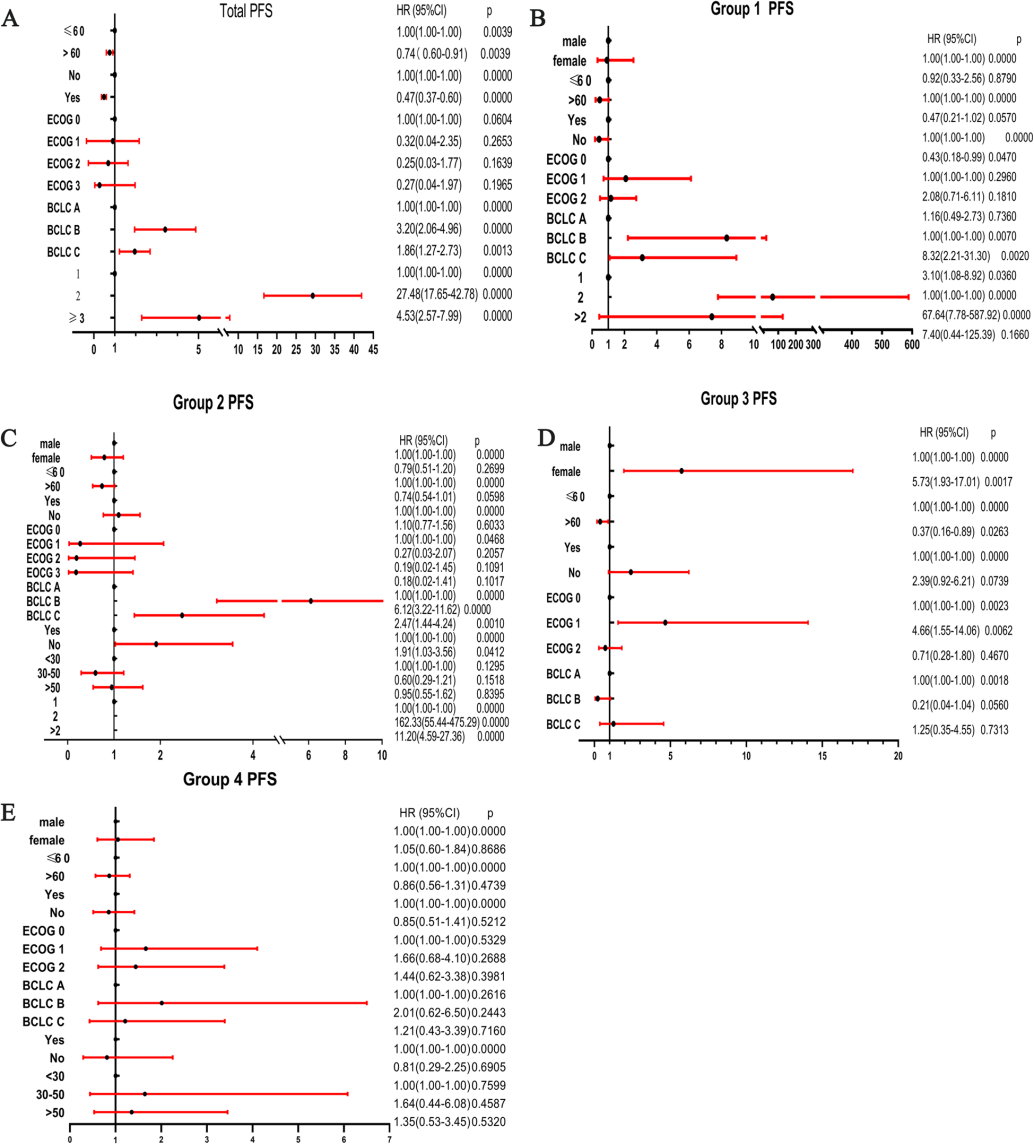
The overall and grouping-related factors of PFS, including overall analysis and grouping analysis.Group1 = Normal weight HCC patients without HBV infection; Group2 = Normal weight HCC patients combined HBV infection; Group3 = Overweight HCC patients without HBV infection; Group4 = Overweight HCC patients combined HBV infection; PFS = progression-free survival; HBV = hepatitis B virus; ECOG = Eastern Cooperative Oncology Group score; BCLC=Barcelona Clinic Liver Cancer; PVTT = portal vein tumor thrombosis

The PFS of each group was analyzed and compared, and the results are shown in Fig. 4. The following potential risk factors for PFS were analyzed in univariate analysis: age, sex, cirrhosis, Milan criteria, Child–Pugh and BCLC classification, tumor diameter and number, tumor location, portal hypertension, and PVTT. The impact of these factors on PFS is summarized as a forest map (Fig. 4).

### Multiple risk factors of OS

**Fig. 5:**
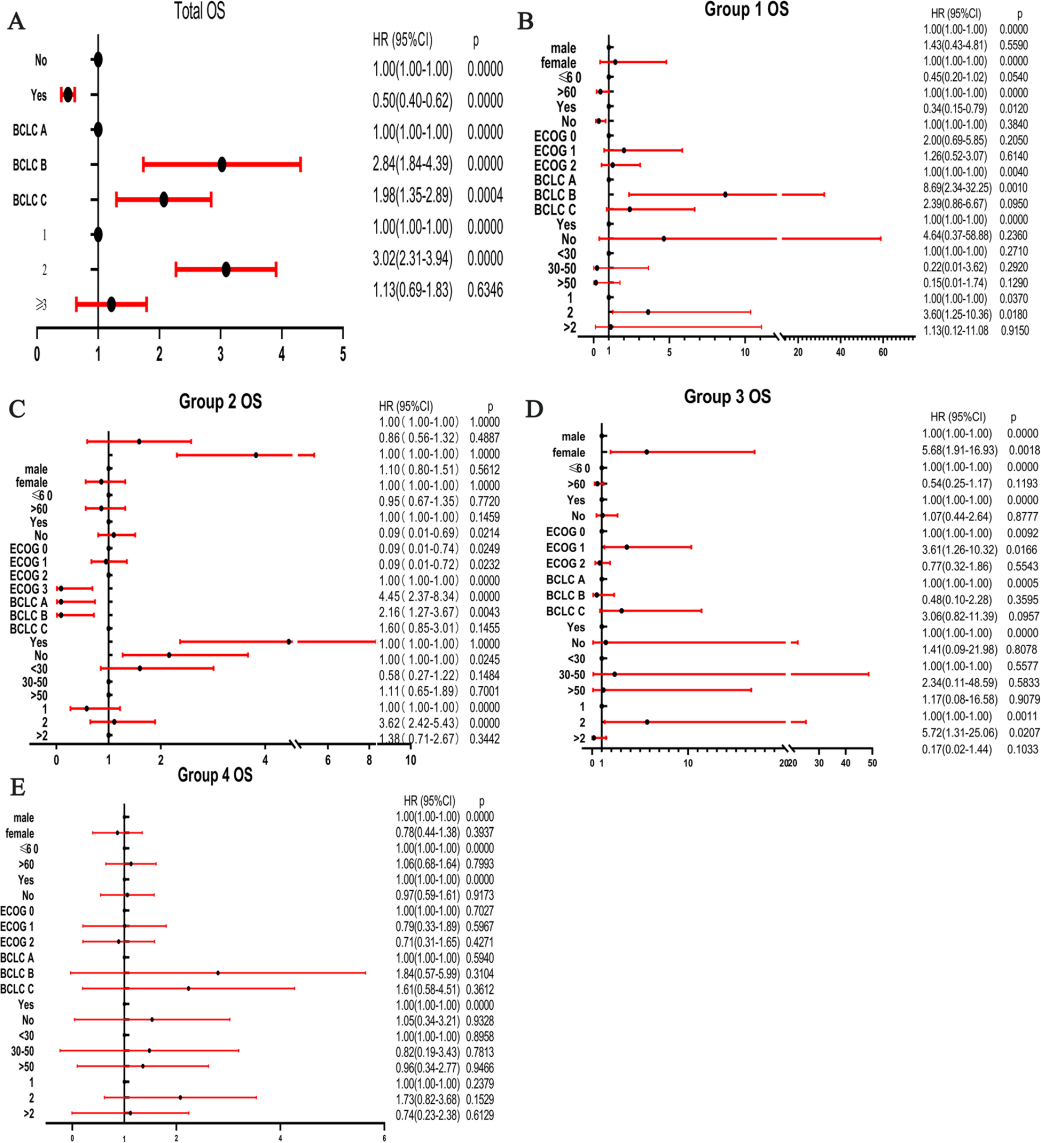
The overall and grouping related factors of OS, including overall analysis and grouping analysis. The overall and grouping-related factors of PFS, including overall analysis and grouping analysis. Group1 = Normal weight HCC patients without HBV infection; Group2 = Normal weight HCC patients combined HBV infection; Group3 = Overweight HCC patients without HBV infection; Group4 = Overweight HCC patients combined HBV infection; OS = overall survival; HBV = hepatitis B virus; ECOG = Eastern Cooperative Oncology Group score; BCLC=Barcelona Clinic Liver Cancer; PVTT = portal vein tumor thrombosis

The statistical results of OS are shown in Fig. 5, and some differences were noted in overweight and HBV-infected HCC patients. Patients with normal weight had better OS than high BMI people. However, in the subsequent study, multivariate analysis indicated that HBV infection is also a major risk factor for OS.

The following potential risk factors for OS were analyzed in univariate analysis: age, sex, cirrhosis, Milan criteria, Child–Pugh and BCLC classification, tumor diameter and number, tumor location, portal hypertension, and PVTT. The impact of these factors on OS is summarized as a forest map (Fig. 5).

### Analysis of adverse reactions

Nausea, vomiting, abdominal pain, liver injury, and fever are common adverse reactions of TACE. Data analysis (Table 4) did not find any significant differences among the four subgroups.

**Table 4:** Incidence of adverse reactions

	Group 1^a^ (*n* = 57)	Group 2^b^ (*n* = 221)	Group 3^c^ (*n* = 58)	Group 4^d^ (*n* = 107)	***P***
Nausea	26	113	26	46	0.518
Vomit	4	30	5	7	0.172
Abdominal pain	27	87	26	52	0.385
Fever	9	32	20	26	0.003
Acute liver failure	1	0	0	1	0.079
Liver abscess	1	2	1	1	0.915

^a^Group 1: normal-weight patients without HBV

^b^Group 2: normal-weight patients combined HBV

^c^Group 3: overweight patients without HBV

^d^Group 4: overweight patients combined HBV

### Analysis of blood samples

**Fig. 6:**
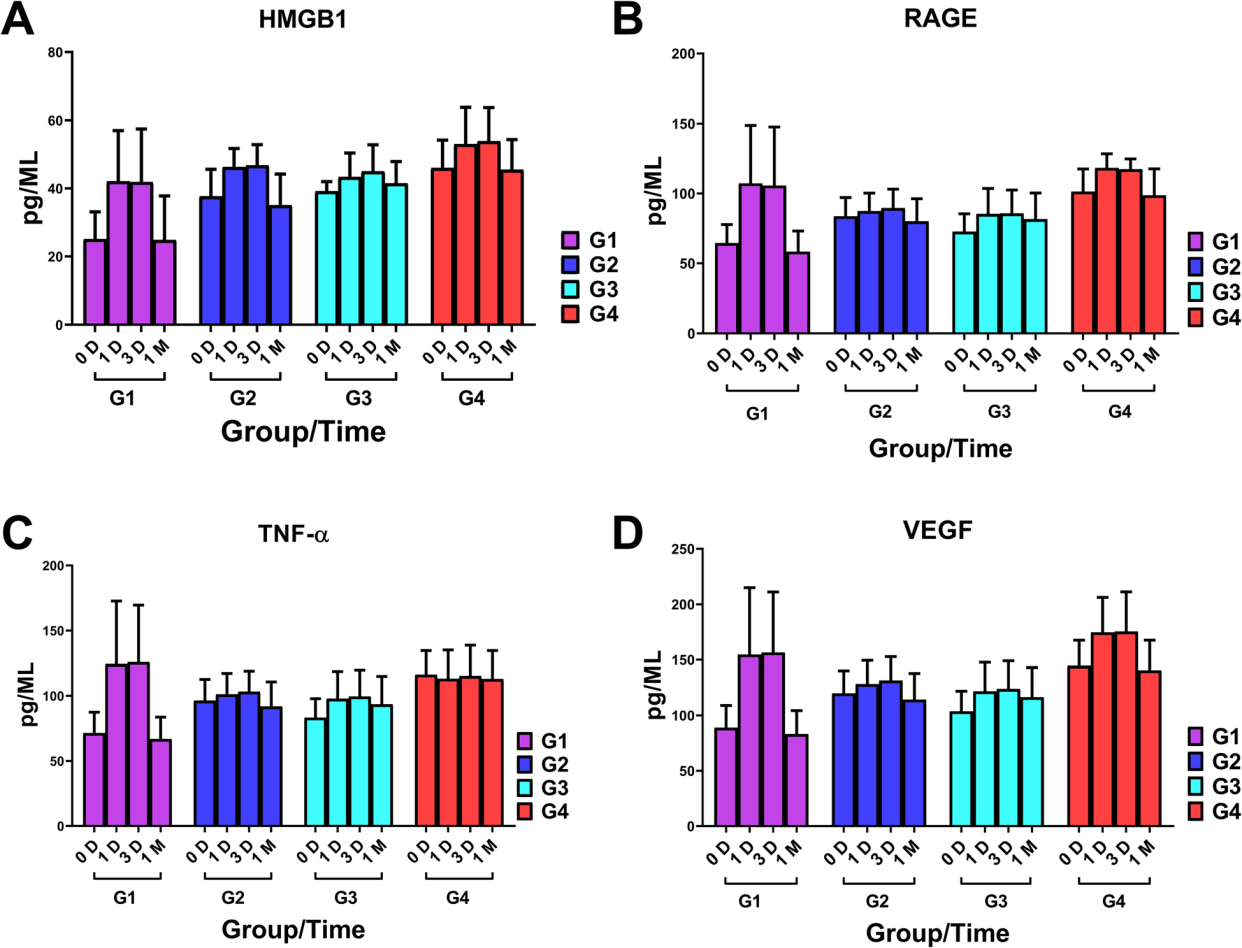
Subgroup analysis of blood samples results according to different population. The concentration analysis results of relevant indicators of HMGB1 in blood samples were compared and analyzed based on the group and time. HMGB1 = high-mobility group protein 1; RAGE = receptor for advanced glycation endproducts; TNF-α = tumor necrosis factor-α; VEGF = vascular endothelial growth factor

The expression of HMGB1, RAGE, TNF-α, and VEGF was increased significantly after TACE. The first patients’ group (normal weight without HBV) had the largest increase on days 1 and 3 (Fig. 6). Before TACE and the first month after the treatment, the expression of HMGB1 in the peripheral blood of patients in the group with HBV infection combined with overweight was higher than the normal weight patients without HBV. Also, no significant differences were observed between overweight alone and HBV alone subgroups, and both groups tended to show similar trends. Although no drastic change was detected in HMGB1 after the treatment in patients with HBV combined overweight, the average expression level was still higher than that of other patients.

### Correlation analysis of HMGB1

**Fig. 7:**
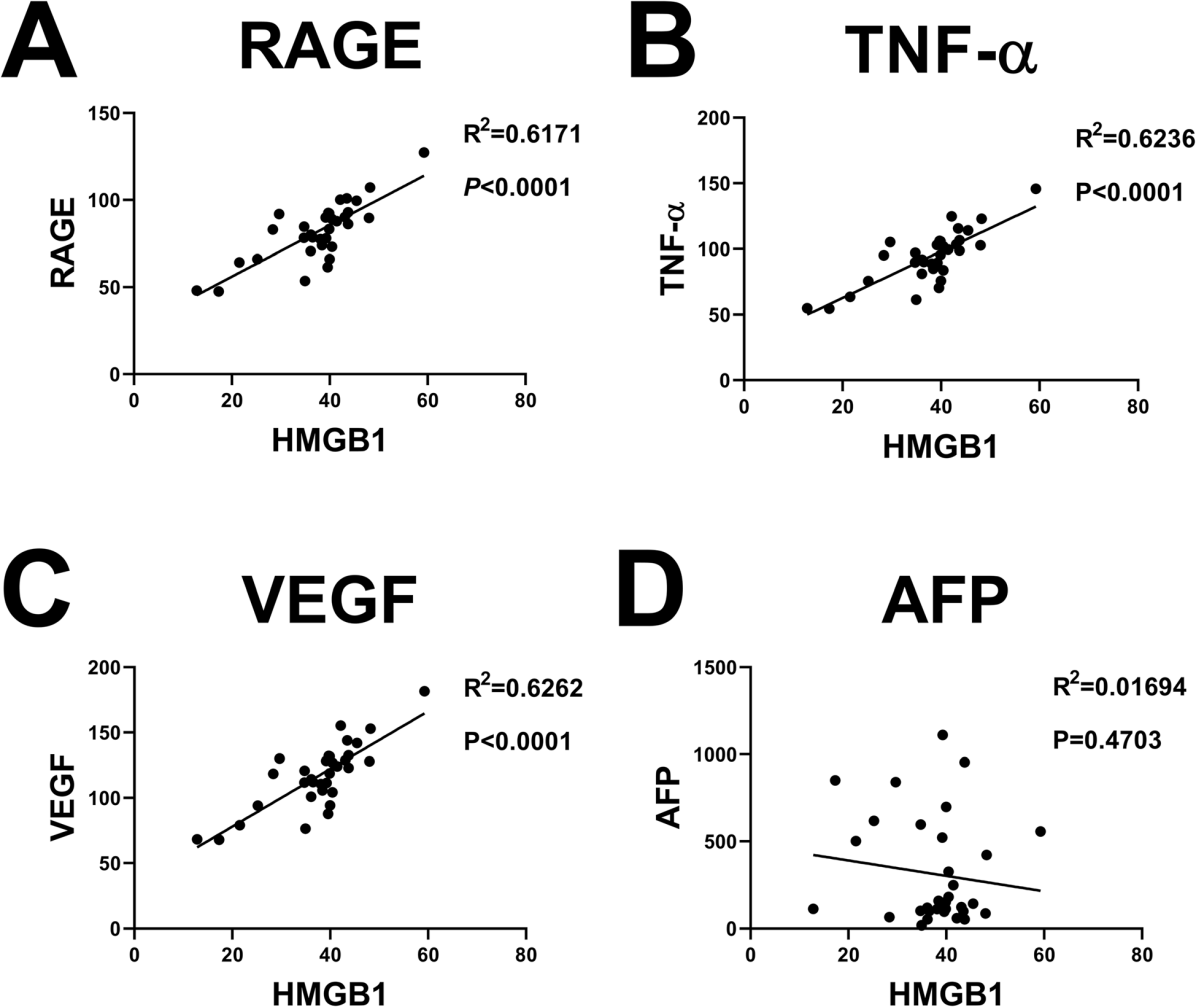
Correlation analysis of HMGB1 and RAGE/TNF-α/VEGF/AFP before TACE.HMGB1 = high-mobility group protein 1; RAGE = receptor for advanced glycation endproducts; TNF-α = tumor necrosis factor-α; VEGF = vascular endothelial growth factor; AFP = alpha fetoprotein

**Fig. 8:**
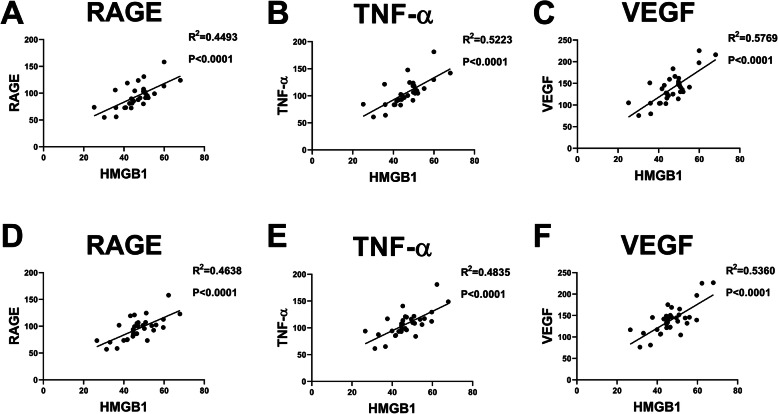
Analysis of the correlation between HMGB1 and RAGE/TNF-α/VEGF/AFP on the days 1 (A/B/C) and day 3 (D/E/F) after TACE. HMGB1 = high-mobility group protein 1; RAGE = receptor for advanced glycation endproducts; TNF-α = tumor necrosis factor-α; VEGF = vascular endothelial growth factor; AFP = alpha fetoprotein

**Fig. 9:**
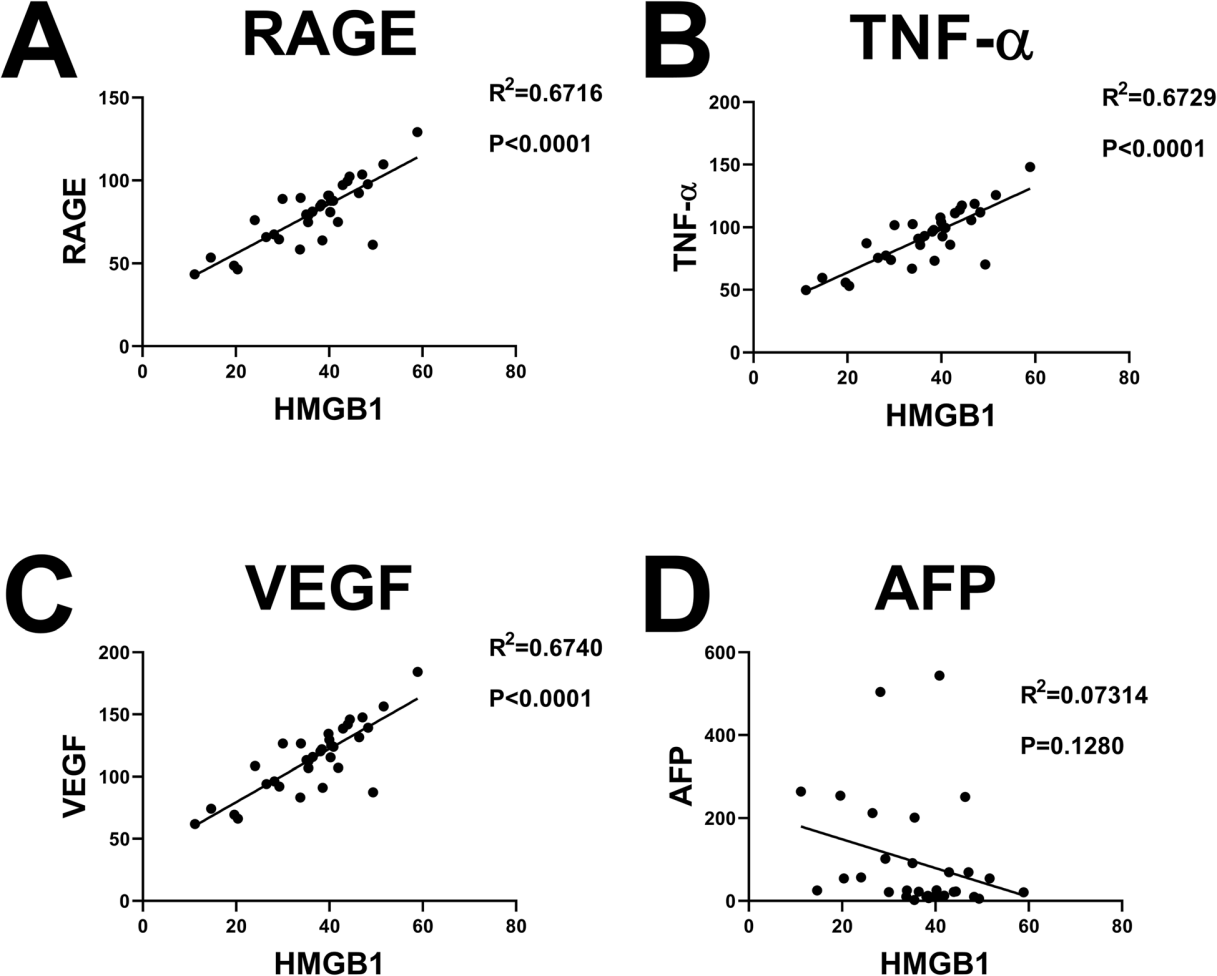
Correlation analysis of HMGB1 and RAGE/TNF-α/VEGF/AFP at 1 month after TACE.HMGB1 = high-mobility group protein 1; RAGE = receptor for advanced glycation endproducts; TNF-α = tumor necrosis factor-α; VEGF = vascular endothelial growth factor; AFP = alpha fetoprotein

The correlation analysis showed a positive correlation among the expression of TNF-α, RAGE, VEGF, and HMGB1 in the four subgroups before and after TACE. The higher the expression of HMGB1, the higher the expression of related indexes or markets. On the other hand, a negative correlation was established between the expression of HMGB1 and tumor marker (alpha-fetoprotein (AFP)). The higher the expression of HMGB1, the greater the change in AFP after the treatment. Figures 7, 8, and 9 suggested that the upregulated HMGB1 indicates an increased expression of TNF-α, RAGE, and VEGF in the blood, and these indexes are positively associated with the development of cancer and negatively correlated with treatment benefits.

**Fig. 10:**
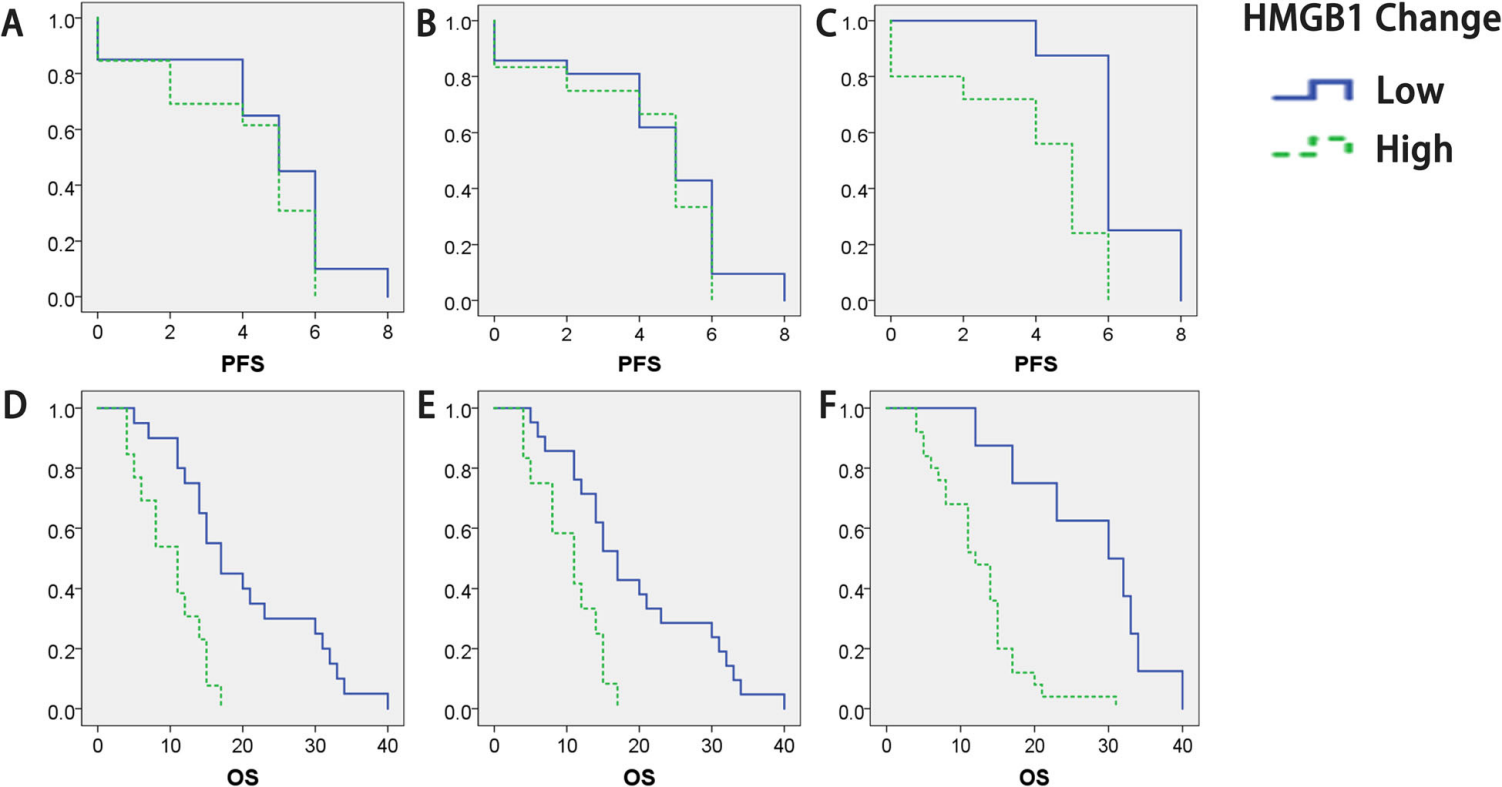
The change of HMGB1 expression level after treatment is related to the patient’s PFS / OS. HMGB1 = high-mobility group protein 1; PFS = progression-free survival; OS = overall survival

PFS and OS were stratified by the low- or high-change of HMGB1 (two-fold greater than or two fold less than that of pre-treatment) and statistically analyzed. Figure 10 shows that increased post-treatment expression of HMGB1 is associated with poor prognosis after TACE.

## Discussion

In this study, the effects of HBV and BMI on PFS, OS, and adverse reactions in patients with HCC were studied. We selected patients who received TACE and compared them to HBV infection or overweight groups. The results showed some differences in PFS and OS among the four subgroups. The PFS/OS of HBV infection group was higher than that of the overweight group. The HBV infection and BMI are crucial for the development of late HCC. Concurrently, we found that the average level of HMGB1 expression in the blood of obese combined HBV-infected HCC patients was higher than that of others, and the expression of HMGB1 was not high in normal weight and non-hepatitis patients. After TACE, the upregulated HMGB1 often occurs in normal-weight people without virus infection, which is also a group of people with better PFS/OS outcomes than others.

In recent years, the correlation between obesity and liver cancer is under intensive focus [14, 15]. Obesity is associated with NAFLD and insulin resistance. Because most overweight or obese patients are NAFLD or NASH, targeting obesity should also focus on the burden of NAFLD. In a retrospective cohort study of NAFLD patients, 490/296707 patients eventually developed HCC, especially fatty cirrhosis [16]. In addition to causing liver injury, obesity-related NAFLD may also promote cancer development [17]. Related studies have shown that several factors of abnormal fat metabolism may lead to the occurrence and development of HCC [18–20].

The primary feature of NAFLD is hepatic steatosis and hepatic lipotoxicity caused by chronic substrate overload [21]. This is a major mechanism in the pathogenesis of NASH and the main risk factor of HCC in overweight people. In addition, the risk of NAFLD includes the effects of DNA damage response (DDR), oxidative stress, immune response, autophagy, and microenvironmental changes [22–24]. The factors aggravate the development risk to HCC in the obese population, forming a complex situation. For example, obesity-induced oxidative stress plays a critical role in T cell protein tyrosine phosphatase (TCPTP) inactivation, leading to interference in STAT-1 and STAT-3 signal transduction and the development of NASH to HCC [25, 26]. Currently, the key role of obesity is explored in the development of cancer, but whether obesity has an impact on the effectiveness of treatment is yet to be elucidated.

Most of the HCC cases in this study are related to HBV infection. Reportedly, about 13–80% of HCC patients have a history of HBV infection, and 30–88% have a history of HCV infection [27–32]. China accounts for one-third of all gastrointestinal cancer cases worldwide, most of which are caused by the high infection rate of *Helicobacter pylori* and HBV/HCV [1, 33, 34]. Therefore, the high proportion of HBV infection could be explained. The common causes of chronic liver cirrhosis are chronic hepatitis infections, alcoholic-caused liver disease, and NASH [35–37]. In developing countries, the lack of effective management of the hepatitis virus often results in liver cirrhosis from chronic hepatitis. The cause of most virus-related HCC cases is the persistent impact of risk factors on the liver in the stage of liver cirrhosis [38–40]. The majority of the HCC patients have a long history of chronic viral hepatitis for at least 10 years. After confirming the long history of hepatitis virus infection and excluding excessive alcohol consumption, long history of fatty liver, and aflatoxin poisoning, we speculated that these were HBV-related HCC cases.

Although the factors inducing HCC in developing countries are different from those in developed countries, diet, work stress, living habits, overweight, and obesity are common in developing countries. Currently, the gap in the obesity rates between low- and middle-income countries and the high-income regions is narrowing [39, 41, 42]. Although there is a lack of large-scale statistical analysis on the weight of all adults, according to the weight statistics of Chinese children and adolescents aged 6–17 years in 2012, the overweight and obesity rates are 9.6 and 6.4%, respectively [43]. Supposedly, this change has clinical implications because high BMI from childhood can cause severe consequences, including type 2 diabetes mellitus, hypertension, cardiovascular disease, and some types of cancer [44, 45]. Moreover, the proportion of overweight children in China is rising rapidly. Although the risk of HCC caused by NAFLD may be lower than that of HCC caused by chronic viral hepatitis, the prevalence of NAFLD-related HCC is sufficient to cause anxiety in the health sector of the developing country. The government has taken several measures to control the spread of the hepatitis virus over the past half-century. The use of large-scale immunization agents and vaccines has decreased the prevalence of HBV/HCV and the number of patients with chronic viral hepatitis [46–48]. The rapid increase in the obesity rate and the decrease in the number of patients with chronic viral hepatitis increased the proportion of overweight-related HCC cases. It is estimated that metabolic syndrome and overweight-related liver diseases affect more than half of the adults in both developed and developing countries in 2030 [48]. According to a meta-analysis of data from 45 studies, the prevalence of NAFLD in the global population increased from 15 to 25.24% in 2005 [49]. Concurrently, a large medical database study conducted by Sanyal showed that NAFLD and NASH are the most common risk factors for HCC patients in the USA conducted by Sanyal [50]. Obese patients are at a high risk of developing liver cancer accompanied by advanced NASH, fibrosis, or cirrhosis. Chronic metabolic abnormalities of hepatocytes include endoplasmic reticulum (ER) and oxidative stress caused by lipotoxicity and necrotizing inflammation, resulting in cell injury or cell death, local inflammation, and fibrosis in the liver [51]. Another result is that compared to HBV/HCV-related liver cancer, which requires a long period of cirrhosis, NAFLD may develop into cancer without cirrhosis. In some patients with NASH-related HCC (usually overweight), liver fibrosis and HCC may not be inseparable [24, 52]. In patients with simple steatosis, overweight may have a high risk of HCC. The progression to cirrhosis in overweight individuals is frequent among middle-aged (40–50-years-old) and elderly. Some studies have shown that NAFLD in obese children is associated with an increased risk of liver cirrhosis in adulthood and the risk of liver cirrhosis and HCC [53]. The problem of obesity is related to income and social development. Even in some low-income developing countries, 1–2% of HCC cases could be attributed to be caused by NAFLD [54]. However, some investigators showed that the actual proportion of HCC patients is higher than estimated because researchers may have ignored the cryptogenic cirrhosis previously. Therefore, like developed countries, developing countries also need to pay more attention to overweight patients.

The results of PFS analysis revealed that normal-weight patients without HBV infection performed best, overweight patients had similar PFS and HBV-related patients, and those with both HBV and high BMI were the worst. The following factors may explain the results. First, changes in liver metabolism caused by HBV and obesity are critical to the development of liver disease. For example, changes in the peroxisome proliferator-activated receptor-gamma coactivators (PGC1) at the transcriptional level give rise to different aspects of liver metabolism, such as oxidative phosphorylation of mitochondria, fatty acid synthesis, and gluconeogenesis. Hepatic steatosis is caused by abnormal mitochondrial and lipid metabolism, which is characterized by the occurrence and development of NAFLD, NASH, and HCC in overweight people. Thus, there could be a link between abnormal PGC1 pathways and the onset of these pathological conditions [55]. Second, abnormal fat metabolism promotes tumor growth by affecting the immune environment. Studies on mouse models and humans have shown that the disorder of lipid metabolism in NAFLD leads to the selective loss of CD4+ in the liver, thereby accelerating the development of HCC [56, 57]. The regulation of abnormal lipid metabolism by agents, such as metformin, might reduce the risk of malignant lesions in obese people. Some metformin targets, such as hexokinase-2 and oxoglutarate dehydrogenase-like (OGDHL), are also regarded as potential biomarkers and therapeutic targets of HCC [58, 59]. These findings suggested that metformin and other drugs improve the survival rate of obese patients by interference with liver metabolism; even a slight improvement in patients could have an enormous impact and save patient lives.

In the analysis results, the tumor characteristics of patients are critical factors in the choice of treatment. A 20-year Italian study showed that the median OS of BCLC B stage patients is 25 months, and TACE (37 months OS) was much longer than surgery or percutaneous treatment (27 months and 36 months, respectively) [60]. BCLC, Child–Pugh grade, and AFP are independent predictors of survival after TACE [61, 62]. However, recent studies on BCLC stage C patients have shown that the advantages and disadvantages of surgery and TACE are yet controversial [63].

Interestingly, the OS of overweight patients seemed to be better than that in HBV infection. This phenomenon could be attributed to the following reasons. First, the number of cases included in the study was small to obtain representative results. Second, the high BMI group consisted of few patients, and hence, the deviation was large, affecting the accuracy of the results. Third, the obesity paradox suggested that patients classified as overweight or obese by BMI have a survival advantage [64]. The risk of cancer is caused by different factors and the prognosis might be carried. Finally, several patients had a prolonged survival time in the high BMI group, which needs further investigation.

In the analysis of common adverse reactions, such as nausea, vomiting, and abdominal pain after TACE, no significant differences were detected between the two groups. These reactions might be related to the patient’s sensitivity to treatment and the dose of chemotherapeutic drugs. The results of the analysis did not detect any difference in the tolerance due to various causes. In addition, no serious adverse reactions, such as treatment-related death or acute liver failure, were observed after all TACE procedures. The results suggested that serious adverse reactions in patients with HCC after TACE might not be common. We also verified the conclusions of other studies that c-TACE and DEB-TACE are safe for patients with advanced cancer.

HMGB1 is the main ligand of RAGE and could be released by tumor cell death after local liver therapy can lead. HMGB1 triggers cell migration and angiogenesis by interacting with RAGE in inflammatory cells, leading to the recruitment of inflammatory cells and the release of mediators in the local inflammatory response. This binding could also stimulate the proliferation and differentiation of cancer cells.

The analysis of blood samples revealed that the content of HMGB1 in the peripheral blood of HCC patients with HBV infection and high BMI is higher than that of other patients before and after treatment, indicating a significant increase in the expression level of HMGB1 when the patients attained local inflammatory reaction. However, after receiving TACE, the level of HMGB1 in patients with inflammatory reaction (viral or steatohepatitis) was stable and did not increase sharply. The expression of RAGE, TNF-α, and VEGF was proportional to that of HMGB1, coupled with the mediating effect of HMGB1 on the local immune verification in the process of hepatic ischemia-reperfusion (I/R). This phenomenon indicates a correlation between the expression of HMGB1 and the curative effect of TACE in HCC.

## Conclusions

Nevertheless, the present study has some limitations, and hence, the results were interpreted cautiously. Some clinical or pathological data were ignored, which might have a potential impact on the long-term prognosis of HCC. Although there was no evidence of a specific correlation between etiology and efficacy, additional studies are required to deepen the understanding of this phenomenon. Taken together, the HBV infection and overweight were associated with a reduced response to TACE, which might be achieved through the upregulated expression of HMGB1.

## Data Availability

All data generated or analysed during this study are included in this published article. The more information that support the findings of this study are available from Shandong Cancer Hospital and Institute Affiliated Shandong First Medical University and Shandong Academy of Medical Sciences but restrictions apply to the availability of these data, which were used under license for the current study, and so are not publicly available. Data are however available from the authors upon reasonable request and with permission of Shandong Cancer Hospital and Institute Affiliated Shandong First Medical University and Shandong Academy of Medical Sciences.

## Abbreviations

HCC: Hepatocellular carcinoma
HBV: Hepatitis B virus
BMI: Body mass index
NAFLD: Nonalcoholic fatty liver disease
NASH: Nonalcoholic steatohepatitis
TACE: Transarterial chemoembolization
PFS: Progression-free survival
OS: Overall survival
ELISA: Enzyme-linked immunosorbent assay
HMGB1: Human high mobility group 1
RAGE: Receptor of advanced glycation end products
TNF-α: Tumor necrosis factor-alpha
VEGF: Vascular endothelial growth factor
